# Socio-demographic, migratory and health-related determinants of food insecurity among Venezuelan migrants in Peru

**DOI:** 10.1017/S1368980023002513

**Published:** 2023-12

**Authors:** Ali Al-kassab-Córdova, David Villarreal-Zegarra, Guido Bendezu-Quispe, Pamela Robles-Valcárcel, Percy Herrera-Añazco, Vicente A. Benites-Zapata

**Affiliations:** 1 Escuela de Medicina, Universidad César Vallejo, Trujillo, Peru; 2 Escuela de Psicología, Universidad Continental, Lima, Peru; 3 Escuela Profesional de Medicina Humana, Universidad Privada San Juan Bautista, Filial de Ica, Ica, Peru; 4 Facultad de Ciencias de la Salud, Universidad Peruana de Ciencias Aplicadas, Lima, Peru; 5 Universidad Privada del Norte, Trujillo, Peru; 6 Maestría en Epidemiología Clínica y Bioestadística, Universidad Científica del Sur, Lima, Peru

**Keywords:** Food insecurity, Venezuela, Transients and migrants, Peru

## Abstract

**Objective:**

To evaluate the factors associated with food insecurity (FI) among Venezuelan migrants residing in Peru. Secondarily, to evaluate the psychometric properties of the Food Insecurity Experience Scale (FIES).

**Design:**

A cross-sectional study based on secondary data analysis of the 2022 Venezuelan Population Residing in Peru Survey (ENPOVE-2022, from the Spanish acronym) was conducted. FI was measured with the FIES, whose properties were tested using the Rasch model. Multinomial logistic regression was performed to estimate relative prevalence ratios with their corresponding 95 % confidence intervals.

**Setting:**

This survey was conducted in February and March 2022 in the eight cities most populated by Venezuelan migrants and refugees in Peru.

**Participants:**

Venezuelan migrants and refugees over the age of 18 years living in Peru.

**Results:**

A total of 7727 participants were included. Rasch reliability was adequate (0·73). The prevalence of mild, moderate and severe FI was 36·71 %, 31·14 % and 10·48 %, respectively. Being aged 25–34 and 35–44 years, unemployed, uninsured, having no formal education or secondary, illegal status, living in a dwelling with 2–4 and more than 4 people, presenting one or more than one chronic disease, residing in Peru for 0–6 months and perceived discrimination were associated with a higher probability of moderate FI. Furthermore, having secondary education, being unemployed, uninsured, never married, illegal, residing in Tumbes, presenting one or more than one chronic disease and perceived discrimination were significantly associated with severe FI.

**Conclusion:**

Four out of ten Venezuelan migrants residing in Peru presented moderate to severe FI. The FIES showed adequate psychometric properties. Differences in the socio-demographic, health and migratory factors associated with FI levels were found. Inter-sectoral and multi-sectoral interventions are needed and should be focused on addressing the determinants of FI.

Food insecurity (FI) is a complex and dynamic process characterized by uncertain or limited access to sufficient nutritious food for an active and healthy life^(1,2)^. FI represents a significant global burden, with the prevalence of moderate to severe FI increasing substantially from 22·7 % in 2016 to 29·3 % in 2021^(3)^. FI represents a public health issue that is strongly associated with a range of adverse health outcomes, including poor sleep quality and quantity^(4)^, stress and anxiety^(5)^, self-reported high blood pressure^(6)^ and cognitive problems^(7)^. Treating these conditions could impose a substantial and enduring economic burden on health systems^(8)^.

FI varies depending on the circumstances in which it is evaluated. Thus, during the pandemic, FI occurred in 75·7 % of the general population in Latin America and the Caribbean, with variations between countries^(9)^. Additionally, FI varies according to some population characteristics, with the status of refugees and migrants being particularly important. In this population, the prevalence of FI varies between 22 % and 70 % depending on the country of origin and the host country^(8,10–12)^. Over the last two decades, the number of international migrants worldwide, including refugees, who account for about 10 % of international migrants, has increased from 174 million in 2000 to 272 million in 2019^(10)^. Due to the increasing number of migrants and refugees, FI in these groups is a concern for international aid agencies and governments^(8,10,13)^. Although more than half of all international migrants worldwide are hosted in high-income countries, many refugees are hosted in low- and middle-income countries^(8)^.

Haiti and Venezuela are two countries of the American continent in which internal political and economic convulsions produced the exodus of their citizens^(14,15)^. Venezuelan migration is the most significant of these two countries, with more than five million migrants arriving at the main destinations of Colombia and Peru. These fluxes have led to various political, social, economic and health problems that have forced governments to establish mitigation measures^(16)^.

Peru is a middle-income country with structural health problems. During the pandemic, Peru reported one of the highest prevalences of FI in the Americas^(9,17)^ and has received more than one million Venezuelan migrants to date^(18)^. Venezuelan migrants in Peru face discrimination, underemployment and lack of access to health insurance^(19)^, all of which may be associated with FI, as shown by some studies on migrants from other countries^(8,10,12)^. However, to the best of our knowledge, only one study evaluated FI in Venezuelan migrants with a validated instrument. This study found that in Trinidad and Tobago, about six in ten respondents exhibited behaviours characterised as severe FI, being less likely among migrants who were employed and more likely among migrants who paid rent^(20)^. Given that the prevalence and factors associated with FI may vary depending on the host countries, their assessment is paramount to identify high-risk groups in which governmental policies could be implemented^(8)^. Therefore, this study aimed to assess the determinants of FI among Venezuelan migrants residing in Peru and, secondarily, to evaluate the psychometric properties of the Food Insecurity Experience Scale (FIES).

## Methods

### Data and setting

**Fig. 1:**
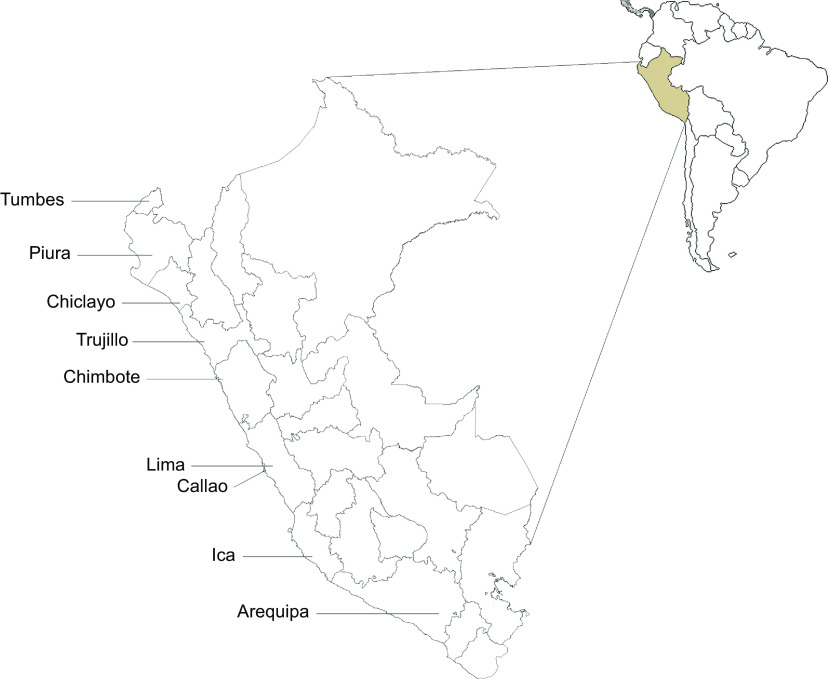
Map of the cities surveyed

This was a cross-sectional study based on secondary data analysis of the 2022 Venezuelan Population Residing in Peru Survey (ENPOVE-2022, from the Spanish acronym). The survey was conducted by the National Institute of Statistics and Informatics (INEI, from the Spanish acronym) in February and March 2022 in the eight cities most populated by Venezuelan migrants and refugees: Lima and Callao, Arequipa, Chiclayo, Chimbote, Ica, Piura, Tumbes and Trujillo (Fig. 1). These provincial capitals had the highest number of dwellings with the Venezuelan population at the national level, which account for 82·9 % of the total population. A total of 138 questions were collected by direct interviews and recorded using digital tablets. The survey covered various aspects of the Venezuelan population residing in Peru, including housing, household and individual characteristics, migration status, health, education, employment, discrimination, gender and victimisation factors^(21)^.

### Sample and inclusion criteria

The ENPOVE-2022 target population included all Venezuelans typically residing in private and collective dwellings in urban areas. The sample frame was generated using data from the National Labour Market Survey and the National Superintendence of Migration. The sampling units were dwellings with the Venezuelan population, households in these dwellings and Venezuelan residents in these dwellings. The sampling design was probabilistic, independent and stratified for each city. People over the age of 12 were interviewed, and the head of the household completed the survey for the youngest. Participants under 18 and adults provided their assent and consent, respectively, whereby it was specified that their participation was voluntary, and they could withdraw at any time. The total sample size was 3680 households. More information about the survey can be found elsewhere^(21)^.

For the current study, we included Venezuelan migrants and refugees living in Peru who were over 18 years of age (the age of majority in Peru) and had complete information on the variables of interest.

### Food insecurity

The outcome variable, FI, was measured through the FIES, which was developed by the FAO to propose a global standard for monitoring hunger worldwide^(22)^. It is an 8-item scale that can be measured at the individual or household level with two reference periods: 30 d or 12 months. The ENPOVE-2022 implemented the survey at the household level in the 30 d preceding the survey. Since the FIES fulfilled the Rasch model assumptions – infit and outfit statistics – a raw score was calculated based on the sum of affirmative responses. The severity of FI was classified as 0 = food security, 1–3 = mild FI, 4–6 = moderate FI and 7–8 = severe FI. The categorisation of the FIES with similar cut-off points has been performed in previous studies^(23–26)^. In addition, it constitutes indicator 2.1.2 of the Sustainable Development Goals^(27)^. The questionnaire was applied in Spanish (Table 1).

**Table 1:** Food Insecurity Experience Scale (FIES)*

Order	Questions	Item	Possible answers
In the last month, have you or any adult member (aged 18 and over) of your household experienced the following situations due to a lack of money or other resources? (En el mes anterior ¿usted o algún miembro adulto (de 18 años y más de edad) de su hogar, debido a la falta de dinero u otros recursos ha pasado por las situaciones siguientes?)			
Q1	Have you felt very worried about not having enough food to eat? (Se han sentido muy preocupados por no tener suficientes alimentos para comer?)	WORRIED	1 = Yes/Si 0 = No/No
Q2	Were you able to eat healthy and nutritious food or food of your choice? (¿Pudieron comer alimentos saludables y nutritivos o de su preferencia?)	HEALTHY	1 = Yes/Si 0 = No/No
Q3	Did you eat only a few types of food? (¿Comió solo unos pocos tipos de alimentos?)	FEWFOODS	1 = Yes/Si 0 = No/No
Q4	Did you have to skip a meal due to lack of food? (¿Tuvo que saltarse una comida por falta de alimentos?)	SKIPPED	1 = Yes/Si 0 = No/No
Q5	Did you eat less than you thought you should? (¿Comió menos de lo que pensaba que debería/n comer?)	ATELESS	1 = Yes/Si 0 = No/No
Q6	Did your household run out of food? (¿Su hogar se quedó sin alimentos?)	RANOUT	1 = Yes/Si 0 = No/No
Q7	Did you feel hungry, but did not eat because you did not have enough money to feed yourself? (¿Tenía hambre, pero no comía por no tener suficiente dinero para alimentarse?)	HUNGRY	1 = Yes/Si 0 = No/No
Q8	Did you go without food for a whole day? (¿Estuvo sin comer durante un día entero?)	WHLDAY	1 = Yes/Si 0 = No/No

* Translation of the FIES was carried out by the INEI team. The FIES questions in Spanish used in the ENDES Survey are provided within parentheses.

The INEI team translated the FIES, which is very similar to the official Spanish translation proposed by FAO^(28)^. However, to enhance the understanding of the scale, item 2 (‘Were you able to eat healthy and nutritious food or food of your choice?’) was modified to a negative item. Consequently, when conducting the analyses, we considered the reversed direction of item 2.

### Covariates

Participant age was divided into six categories: 18–24, 25–34, 35–44, 45–54, 55–64 and 65 years or older. The level of education attained in Peru or Venezuela was categorised as no formal education or primary, secondary or higher. In addition, the presence of chronic diseases – arthritis, hypertension, asthma, rheumatism, diabetes, tuberculosis, hypercholesterolaemia, heart disease, lung disease, cancer, mental disease, HIV/AIDS and other sexually transmitted diseases, among others – was categorised as none, one and more than one. The number of residents in the dwelling was grouped as 1, 2–4 and more than four people. Marital status was also classified as never married, currently married and previously married. Other variables included were sex (male/female), socio-economic status (low/middle/high), having a mental or physical disability (yes/no), employment status (unemployed/employed), health insurance (uninsured/insured) and perceived discrimination (yes/no).

### Statistical analysis

#### Rasch model

We used the Rasch model to examine the psychometric properties of the FIES. All analyses were performed with the ‘RM.weights’ package in RStudio (RStudio Team (2020). RStudio: Integrated Development for R. RStudio, PBC. URL http://www.rstudio.com/) using the guidelines of the FAO^(29)^. To compare our results with other studies, we followed the standard procedure of excluding extreme raw scores (zero and eight points) to avoid possible bias resulting from a large proportion of these raw scores^(24,30)^. In addition, we assessed the Rasch assumptions that items discriminate equally and are conditionally independent and unidimensional^(24)^. The Rasch model transforms ordinal raw scores into continuous data with equal interval units (logits) that indicate the severity of the latent trait as measured by the raw scores, allowing for the summation of raw scores. In the analysis of the FIES, the difficulty and discrimination parameters were estimated for each item to assess the scale’s psychometric properties. The difficulty parameter represents the level of the trait being measured – such as FI – required to endorse a particular item. In other words, it reflects the trait level at which an individual is equally likely to endorse or not endorse an item. The discrimination parameter reflects the item’s ability to differentiate between individuals with different levels of the trait being measured. A high discrimination parameter indicates that an item can distinguish well between individuals with different levels of the trait. In contrast, a low discrimination parameter indicates that an item may not be able to distinguish well.

Rasch modelling outputs also include the calculation of infit and outfit statistics. Infit statistics are used to evaluate the fit of individual items within the item response theory model. A value close to 1 indicates a good fit, while values less than 0·7 or greater than 1·3 indicate a poor fit. Outfit statistics are used to evaluate the fit of the entire item response theory model. A value close to 0 indicates a good fit, while values greater than 3 indicate a poor fit^(31)^.

We conducted a modified Rasch reliability analysis. Values were considered adequate if they were greater than 0·70. We also assessed the correlations of the remaining items. If the residual correlations are large and positive (>0·40), it suggests that the items measure a common underlying construct and are, therefore, conditionally dependent. On the other hand, if the residual correlations are small or close to zero, it suggests that the items are measuring distinct constructs and are, therefore, conditionally independent (possible multidimensionality)^(30,32)^. Rasch models were used instead of a classical test theory approach because the original FIES design is based on Rasch models, and their manual strongly recommends using Rasch models for validating the scale^(33)^. In addition, we calculated the following fitness indices for the FIES: Tucker-Lewis Index (TLI), Comparative Fit Index (CFI), Standardised Root Mean Square Residual (SRMSR) and Root Mean Square Error of Approximation (RMSEA).

#### Descriptive, bivariate and multinomial logistic regression analyses

The data analysis was performed in Stata 16.0 (Stata Corp.) with the *svy* package for complex surveys. Absolute and relative frequencies were estimated to describe the sample. The Chi-square test with Rao–Scott correction was employed for the bivariate analysis to test for potential associations. Variables with a *P*-value < 0·05 were included in the regression analysis. We employed a multinomial logistic regression model given that the outcome variable (FI) consisted of more than two categories: food security, mild FI, moderate FI and severe FI. To assess the association, relative prevalence ratios (RPR) and their corresponding 95 % CI were reported. *P*-values < 0·05 were considered statistically significant.

### Ethical considerations

This study did not require the approval of an ethics committee to be conducted as it was a secondary analysis of the ENPOVE, whose data are in the public domain and do not allow the identification of the evaluated participants. The survey and data can be accessed from the INEI website (https://proyectos.inei.gob.pe/microdatos/). The ENPOVE interviewers obtained the prior consent of the respondents to participate in the study.

## Results

### Description of the sample

A total of 7727 participants were included (see flowchart in online Supplemental Fig. 1). Table 2 shows the socio-demographic and migratory characteristics of Venezuelan migrants living in Peru. The sex ratio was balanced with 51·08 % female. Most respondents (40·85 %) were between 25 and 34 years old. At least half had a higher education, and almost 40 % had a low socio-economic status. Approximately three-quarters were employed and had no health insurance. Most participants had a legal migratory status (70·41 %) and had been residing in Peru for over 12 months (84·55 %). Finally, roughly eight out of ten respondents were single and lived in Lima.

**Table 2:** Socio-demographic, migratory and health-related characteristics of Venezuelan migrants living in Peru

Characteristics	Total		
	Absolute frequency of participants surveyed	Weighted proportion according to each category	
	*n*	%	95 % CI
Socio-demographic factors			
Sex			
Male	4212	48·91	48·00, 49·82
Female	4412	51·08	50·17, 51·99
Age (years)			
18–24	1732	19·34	17·82, 20·96
25–34	3313	40·85	39·28, 42·43
35–44	1780	21·84	20·93, 22·77
45–54	921	10·70	9·71, 11·77
55–64	442	5·11	4·51, 5·78
65 or older	191	2·15	1·64, 2·81
Mean (sd)	34·42 (11·62)		
	*n*	%	
Education level			
No formal education or primary	1022	9·55	8·30, 10·97
Secondary	3445	44·33	41·52, 47·17
Higher	3260	46·10	43·21, 49·02
Socio-economic status			
Lower	3680	39·45	22·25, 59·74
Middle	3043	36·66	17·52, 61·19
Higher	1931	23·87	11·41, 43·30
Employment status			
Employed	5936	77·66	76·40, 78·87
Unemployed	1791	22·34	21·13, 23·60
Marital status			
Never married	6926	82·28	80·73, 83·73
Currently married	1215	15·04	13·63, 16·57
Previously married	238	2·68	2·28, 3·15
Number of residents			
1	975	11	9·89, 12·23
2–4	5278	63·27	60·74, 65·74
More than 4	2371	25·72	23·32, 28·28
City of residence			
Arequipa	472	3·37	2·67, 4·26
Chiclayo	522	1·65	1·30, 2·09
Chimbote	587	1·58	1·20, 2·06
Ica	453	2·40	1·80, 3·20
Lima	4575	82·74	80·66, 84·64
Piura	519	2·25	1·72–2·93
Trujillo	951	4·93	3·96, 6·12
Tumbes	575	1·08	0·80, 1·46
Health-related factors			
Mental or physical disability			
Yes	153	1·98	1·65, 2·37
No	7800	98·01	97·62, 98·34
Health insurance			
Uninsured	6317	75·85	73·64, 77·92
Insured	1636	24·14	22·07, 26·35
Having chronic diseases			
None	7319	85·01	84·05, 85·92
1	1143	12·86	12·04, 13·73
>1	192	2·11	1·75, 2·55
Migratory factors			
Migratory status			
Legal	5043	70·41	67·65, 73·02
Illegal	2910	29·59	26·98, 32·35
Time residing in Peru (in months)			
0–6	787	9·09	8·30, 9·95
7–12	558	6·36	5·65, 7·16
More than 12	6608	84·55	83·45, 85·58
Perceived discrimination			
Yes	2634	33·47	31·71, 35·27
No	5093	66·53	64·73, 68·29

### Fit statistics and overall reliability of Food Insecurity Experience Scale

The FIES presented acceptable infit and outfit values, which adequately approximate the Rasch model’s assumption of equal discrimination. Nevertheless, item 2 presented high values for infit. The items with the highest affirmative response rates were ‘WORRIED’, ‘FEWFOODS’ and ‘ATELESS’ (see Table 3). Rasch’s reliability was adequate (0·73). Additionally, all residual correlations were between -0·4 and 0·4 for each pair of items. The equating for comparability is shown in online Supplemental Table 1. Finally, the overall fit of the FIES to the Rasch model was good (CFI = 0·989; TLI = 0·984; SRMSR = 0·028; RMSEA (90 %CI) = 0·050 (0·046, 0·054)).

**Table 3:** Proportion of affirmative responses to FIES items, severity and item fit statistics

*n*	Item	Affirmative responses	Item severity	Item SE	Infit	Outfit
1	WORRIED	62·25	2·055	0·029	0·880	0·813
2	HEALTHY	32·24	−0·345	0·027	1·708	2·805
3	FEWFOODS	52·37	1·324	0·027	1·060	1·189
4	SKIPPED	39·35	0·178	0·026	0·834	0·718
5	ATELESS	43·77	0·598	0·026	0·749	0·656
6	RANOUT	24·85	−0·930	0·028	0·876	0·780
7	HUNGRY	33·25	−0·277	0·027	0·738	0·574
8	WHLDAY	9·43	−2·603	0·039	0·984	1·506

FIES, Food Insecurity Experience Scale.

### Food insecurity

**Fig. 2:**
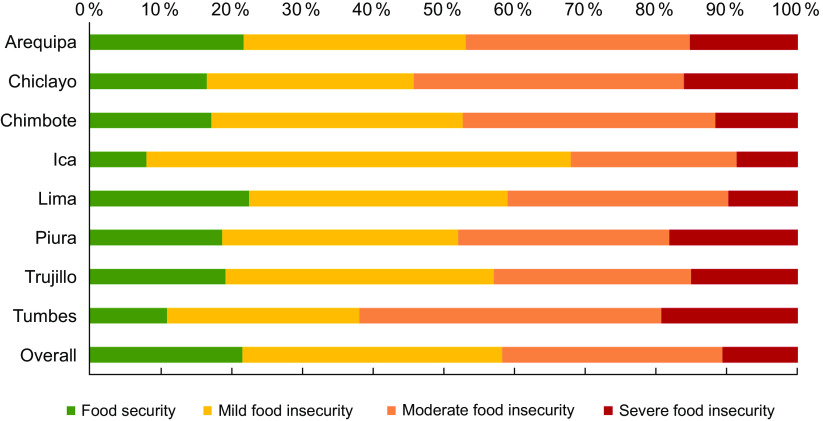
Food insecurity among Venezuelan migrants according to the cities surveyed

Figure 2 describes the prevalence of FI according to each city. Nationwide, the prevalence of mild FI was 36·71 % (95 % CI(34·36, 39·11)), moderate FI 31·14 % (95 % CI(28·89, 33·49)) and severe FI 10·48 % (95 % CI(9·17, 11·97)). The highest prevalence of severe FI was in Tumbes (19·13 %), followed by Piura (18·01 %) and Chiclayo (15·95 %). Contrarily, the city with the highest prevalence of food security for Venezuelan migrants was Lima (22·6 %), followed by Arequipa (12·81 %) and Trujillo (19·25 %).

### Bivariate analysis

Table 4 shows the distribution of mild, moderate and severe FI among Venezuelan migrants according to socio-demographic, migratory and health-related characteristics. Most covariates exhibited statistically significant differences concerning FI, except for socio-economic status (*P* = 0·0523) and having a mental or physical disability (*P* = 0·1126). The highest prevalence of moderate FI was observed among individuals who were previously married (34·47 %), residing with more than four people (34·99 %), based in Tumbes (42·74 %), having a mental or physical disability (42·81 %), having more than one chronic disease (38·38 %), having illegal migratory status (35·28 %), having resided between 7 and 12 months in Peru (48·15 %) and experiencing perceived discrimination (36·29 %). Similarly, the highest prevalence of severe FI was found among subjects aged 18–24 years old (13·48 %), with informal or primary education (13·22 %), unemployed (13·61 %), never married (11·21 %), based in Tumbes (19·13 %), uninsured (11·90 %), with an illegal migratory status (15·56 %), having more than one chronic disease (14·97 %), residing between 0 and 6 months in Peru (16·79 %) and having perceived discrimination (14·46 %).

**Table 4:** Food insecurity according to socio-demographic, migratory and health-related characteristics of Venezuelan migrants living in Peru

Characteristics	No FI		Mild FI		Moderate FI		Severe FI		*P* *
	%	95 % CI†	%	95 % CI†	%	95 % CI†	%	95 % CI†	
Socio-demographic factors									
Sex									0·0022
Male	23·08	20·72, 25·61	36·65	34·14, 39·23	30·09	27·71, 32·58	10·18	8·76, 11·81	
Female	20·32	18·24, 22·56	36·76	34·30, 39·29	32·15	29·71, 34·70	10·77	9·36, 12·37	
Age (years)									0·0306
18–24	22·39	19·00, 26·20	33·24	29·47, 37·23	30·89	27·43, 34·58	13·48	10·91, 16·54	
25–34	22·14	19·57, 24·95	37·37	34·43, 40·41	31·37	28·44, 34·44	9·12	7·61, 10·90	
35–44	19·89	16·88, 23·28	36·46	32·91, 40·17	33·4	29·93, 37·07	10·25	8·24, 12·67	
45–54	22·68	18·89, 26·98	39·29	34·89, 43·87	27·25	23·57, 31·28	10·77	8·26, 13·93	
55–64	18·82	14·19, 24·53	42·33	35·87, 49·05	28·41	23·14, 34·35	10·44	7·36, 14·61	
65 or older	27·56	19·23, 37·81	38·28	28·47, 49·14	25·02	17·73, 34·07	9·14	4·97, 16·21	
Education level									<0·0001
No formal education or primary	15·85	12·05, 20·57	37·7	32·36, 43·35	33·23	28·24, 38·63	13·22	9·71, 17·74	
Secondary	18·57	16·13, 21·29	35·33	32·32, 38·45	33·24	30·38, 36·22	12·86	10·93, 15·08	
Higher	24·73	22·00, 27·67	37·18	34·18, 40·28	29·65	26·77, 32·69	8·45	6·89, 10·32	
Socio-economic status									0·0523
Lower	25·7	22·22, 29·53	35·55	32·33, 38·91	28·22	24·87, 31·83	10·53	8·38, 13·14	
Middle	18	14·85, 21·64	38·23	34·18, 42·45	33·79	29·90, 37·92	9·981	8·06, 12·30	
Higher	20·63	16·75, 25·13	36·27	31·12, 41·75	31·91	27·43, 36·76	11·19	8·60, 14·43	
Employment status									<0·0001
Employed	22·79	20·47, 25·30	36·45	33·99, 38·99	30·68	28·25, 33·24	10·07	8·69, 11·64	
Unemployed	15·44	13·01, 18·22	36·25	32·72, 39·93	34·7	31·39, 38·17	13·61	11·25, 16·37	
Marital status									0·0055
Never married	21·32	19·12, 23·70	35·88	33·35, 38·49	31·59	29·17, 34·11	11·21	9·73, 12·89	
Currently married	24·22	19·85, 29·21	41·73	36·96, 46·66	27·1	22·75, 31·94	6·95	4·98, 9·60	
Previously married	19·26	13·36, 26·96	39·35	31·31, 48·01	34·47	26·91, 42·91	6·91	4·23, 11·11	
Number of residents									0·0137
1	30·03	25·04, 35·53	35·74	31·08, 40·68	25·85	21·61, 30·61	8·38	6·18, 11·28	
2–4	21·9	19·55, 24·45	37·29	34·60, 40·06	30·5	28·00, 33·12	10·31	8·76, 12·08	
More than 4	17·51	13·39, 22·57	35·68	30·44, 41·28	34·99	29·69, 40·70	11·82	8·82, 15·66	
City of residence									<0·0001
Arequipa	21·81	16·18, 28·73	31·41	24·47, 39·30	31·67	24·68, 39·59	15·11	9·89, 22·42	
Chiclayo	16·59	11·13, 24·00	29·28	22·28, 37·42	38·18	30·63, 46·34	15·95	10·70, 23·11	
Chimbote	17·29	11·64, 24·92	35·5	27·85, 43·98	35·74	28·46, 43·73	11·47	6·04, 20·70	
Ica	8·06	3·98, 15·64	60	50·13, 69·12	23·5	16·58, 32·20	8·43	4·78, 14·45	
Lima	22·6	20·13, 25·28	36·56	33·82, 39·39	31·19	28·54, 33·98	9·65	8·17, 11·36	
Piura	18·81	13·51, 25·57	33·32	26·21, 41·27	29·87	22·86, 37·96	18·01	12·40, 25·42	
Trujillo	19·25	14·54, 25·05	37·92	31·92, 44·32	27·92	23·06, 33·35	14·91	9·82, 21·98	
Tumbes	10·97	6·30, 18·42	27·18	20·41, 35·21	42·72	35·04, 50·77	19·13	13·98, 25·61	
Health-related factors									
Mental or physical disability									0·1126
Yes	19·46	12·55, 28·92	29·16	21·00, 38·92	42·81	32·18, 54·16	8·57	4·77, 14·93	
No	21·16	19·03, 23·46	36·45	34·05, 38·91	31·47	29·18, 29·18	10·93	9·53, 12·50	
Health insurance									0·0001
Uninsured	19·53	17·35, 21·91	36·08	33·54, 38·70	32·49	30·02, 35·06	11·90	10·32, 13·69	
Insured	26·13	22·49, 30·14	37·00	33·02, 41·16	29·20	25·58, 33·10	7·68	6·02, 9·75	
Having chronic diseases									0·0001
None	22·67	20·45, 25·05	36·94	34·51, 39·44	30·52	28·20, 32·95	9·87	8·54, 11·38	
1	16·53	13·48, 20·11	35·61	31·59, 39·85	34·05	30·08, 38·25	13·82	11·09, 17·08	
>1	12·75	7·82, 20·13	33·9	25·95, 42·87	38·38	29·76, 47·80	14·97	9·40, 22·99	
Migratory factors									
Migratory status									<0·0001
Legal	23·37	21·00, 25·91	37·53	34·92, 40·22	30·19	27·66, 32·84	8·91	7·49, 10·58	
Illegal	15·78	13·01, 19·02	33·37	29·82, 37·12	35·28	31·87, 38·86	15·56	13·24, 18·21	
Time residing in Peru (in months)									0·0003
0–6	22·68	18·00, 28·16	29·5	24·56, 34·98	31·03	26·08, 36·45	16·79	12·75, 21·80	
7–12	19·84	14·96, 25·83	32·01	26·08, 38·58	32·37	26·30, 39·09	15·78	11·89, 20·65	
More than 12	21·05	18·94, 23·33	37·36	34·89, 39·89	31·72	29·30, 34·24	9·88	8·50, 11·44	
Perceived discrimination									<0·0001
Yes	18·14	15·67, 20·92	31·11	28·16, 34·23	36·29	33·04, 39·66	14·46	12·07, 17·22	
No	22·66	20·11, 25·44	39·07	36·35, 41·87	29·22	26·71, 31·86	9·05	7·67, 10·66	

FI, food insecurity.

* Chi-square test with Rao–Scott correction.

### Multinomial logistic regression analysis

The adjusted regression model showed that several factors were associated with having mild, moderate and severe FI among Venezuelan migrants living in Peru. The factors associated with mild FI were being between 35 and 44 years old (RPR = 1·94; 95 % CI1·04, 3·62)), having secondary education (RPR = 1·26; 95 % CI(1·01, 1·57)), unemployment (RPR = 1·38; 95 % CI(1·10, 1·72)), residing in Ica (RPR = 4·65; 95 % CI(2·16, 10·00)) and having resided in Peru for 0–6 months (RPR = 0·57; 95 % CI(0·41, 0·81)).

The factors associated with a higher frequency of moderate FI in the adjusted analysis were being between 25 and 34 (RPR = 2·07; 95 % CI(1·02, 2·99)) and 35 and 44 years old (RPR = 2·55; 95 % CI(1·27, 5·12)), having no formal education or primary (RPR = 1·52; 95 % CI(1·02, 2·25)) and secondary education (RPR = 1·43; 95 % CI(1·15, 1·77)), being unemployed (RPR = 1·53; 95 % CI(1·19, 1·96)), living with 2–4 residents (RPR = 1·51; 95 % CI(1·08, 2·10)) and more than four residents (RPR = 2·05; 95 % CI (1·28, 3·29)), being uninsured (RPR = 1·32; 95 % CI(1·03, 1·70)), having one (RPR = 1·35; 95 % CI(1·04, 1·75)) or more than one chronic disease (RPR = 2·39; 95 % CI(1·15, 4·95)), having illegal status (RPR = 1·59; 95 % CI(1·22, 2·06)), having resided in Peru for 0–6 months (RPR = 0·69; 95 % CI(0·49, 0·98)) and experiencing perceived discrimination (RPR = 1·66; 95 % CI (1·34, 2·06)).

The factors associated with a higher prevalence of severe FI included having secondary education (RPR = 1·69; 95 % CI (1·24, 2·29)), being unemployed (RPR = 1,81; 95 % CI (1·30, 2·51)), being never married (RPR = 1·69; 95 % CI (1·08, 2·67)), residing in Tumbes (RPR = 2·39; 95 % CI (1·17, 4·90)), lacking insurance coverage (RPR = 1·54; 95 % CI (1·07, 2·19)), having one (RPR = 1·56; 95 % CI (1·10, 2·22)) or more than one chronic disease (RPR = 2·93; 95 % CI (1·32, 6·50), illegal (RPR = 1·89; 95 % CI (1·40, 2·55)) and reporting perceived discrimination (RPR = 2·27; 95 % CI (1·68, 3·05)) (Table 5).

**Table 5:** Factors associated with food insecurity in Venezuelan migrants living in Peru

Variables	Mild FI		Moderate FI		Severe FI	
	RPR†	95 % CI	RPR†	95 % CI	RPR†	95 % CI
Socio-demographic factors						
Sex						
Male	0·97	0·80, 1·04	0·95	0·82, 1·09	1·03	0·85, 1·25
Female	Ref.	Ref.	Ref.	Ref.	Ref.	Ref.
Age (years)						
18–24	1·31	0·67, 2·55	1·46	0·71, 2·99	1·40	0·50, 3·91
25–34	1·80	0·94, 3·43	2·07*	1·02, 2·99	1·55	0·56, 4·28
35–44	1·94*	1·04, 3·62	2·55**	1·27, 5·12	2·13	0·76, 5·93
45–54	1·57	0·82, 2·99	1·55	0·76, 3·13	1·65	0·59, 4·60
55–64	1·94	0·98, 3·84	1·88	0·92, 3·85	1·84	0·70, 4·80
65 or older	Ref.	Ref.	Ref.	Ref.	Ref.	Ref.
Education level						
No formal education or primary	1·44	0·99, 2·09	1·52*	1·02, 2·25	1·62	0·97, 2·71
Secondary	1·26*	1·01, 1·57	1·43**	1·15, 1·77	1·69**	1·24, 2·29
Higher	Ref.	Ref.	Ref.	Ref.	Ref.	Ref.
Employment status						
Employed	Ref.	Ref.	Ref.	Ref.	Ref.	Ref.
Unemployed	1·38**	1·10, 1·72	1·53**	1·19, 1·96	1·81***	1·30, 2·51
Marital status						
Never married	0·96	0·69, 1·33	1·24	0·88, 1·76	1·69*	1·08, 2·67
Currently married	Ref.	Ref.	Ref.	Ref.	Ref.	Ref.
Previously married	1·19	0·64, 2·21	1·62	0·86, 3·05	0·92	0·39, 2·13
Number of residents						
1	Ref.	Ref.	Ref.	Ref.	Ref.	Ref.
2–4	1·23	0·91, 1·64	1·51*	1·08, 2·10	1·35	0·89, 2·07
More than 4	1·41	0·92, 2·15	2·05**	1·28, 3·29	1·73	0·95, 3·13
City of residence						
Arequipa	0·86	0·53, 1·38	0·98	0·59, 1·61	1·44	0·77, 2·71
Chiclayo	0·81	0·46, 1·43	1·25	0·70, 2·22	1·48	0·75, 2·93
Chimbote	1·03	0·61, 1·74	1·08	0·62, 1·88	0·96	0·41, 2·23
Ica	4·65***	2·16, 10·00	1·93	0·87, 4·25	1·90	0·75, 4·77
Lima	Ref.	Ref.	Ref.	Ref.	Ref.	Ref.
Piura	1·13	0·66, 1·91	1·06	0·64, 1·76	1·88	0·99, 3·55
Trujillo	1·19	0·77, 1·82	0·91	0·58, 1·43	1·60	0·83, 3·06
Tumbes	1·18	0·59, 2·36	1·94	0·96, 3·90	2·39*	1·17, 4·90
Health-related factors						
Health insurance						
Uninsured	1·24	0·98, 1·59	1·32*	1·03, 1·70	1·54*	1·07, 2·19
Insured	Ref.	Ref.	Ref.	Ref.	Ref.	Ref.
Having chronic diseases*						
None	Ref.	Ref.	Ref.	Ref.	Ref.	Ref.
1	1·22	0·94, 1·58	1·35*	1·04, 1·75	1·56*	1·10, 2·22
>1	1·60	0·95, 3·09	2·39*	1·15, 4·95	2·93**	1·32, 6·50
Migratory factors						
Migratory status						
Legal	Ref.	Ref.	Ref.	Ref.	Ref.	Ref.
Illegal	1·23	0·94, 1·61	1·59***	1·22, 2·06	1·89***	1·40, 2·55
Time residing in Peru (in months)						
0–6	0·57**	0·41, 0·81	0·69*	0·49, 0·98	1·07	0·70, 1·64
7–12	0·85	0·54, 1·34	0·78	0·49, 1·25	1·33	0·82, 2·18
More than 12	Ref.	Ref.	Ref.	Ref.	Ref.	Ref.
Perceived discrimination						
Yes	1·02	0·83, 1·25	1·66***	1·34, 2·06	2·27***	1·68, 3·05
No	Ref.	Ref.	Ref.	Ref.	Ref.	Ref.

RPR, relative prevalence ratio; FI, food insecurity.

* *P* < 0·05.

† Multinomial logistic regression adjusted per all model variables.

** *P* < 0·01.

*** *P* < 0·001.

## Discussion

### Main findings

This study aimed to identify socio-demographic, migratory and health-related variables associated with FI among Venezuelan migrants in Peru. Previously, we assessed the psychometric properties of the FIES in our sample, which exhibited an adequate model fit. The results revealed that approximately three out of every four participants experienced some type of FI. Differences in the socio-demographic, health status and migrant factors associated with mild, moderate and severe FI were found. In general, unemployment and secondary education were associated with any grade of FI. Being uninsured, having chronic diseases, possessing an illegal migratory status and experiencing perceived discrimination were associated with moderate and severe levels of FI. Moreover, lacking formal education or having only completed primary education, being aged 25–34 or 35–44 years, having a household size greater than one, residing in Ica and having lived in Peru for 0–6 months were all associated with either mild or moderate FI. Finally, being never married and residing in Tumbes were directly associated with the presence of severe FI. It is important to note that the magnitude of the association intensified as the level of FI increased.

### Comparison with previous studies and plausibility of the results

Different studies have identified that the FIES has adequate differential item functioning, infit, outfit and Rasch reliability values^(23,24,34)^. Our study is in line with these findings. However, our study found that item 2 had a low discriminatory capacity and requires revision in further studies. Overall, we identified that the FIES has optimal measurement properties and is useful for assessing FI in the Venezuelan migrant population.

Other studies have also estimated the prevalence of FI in migrants. A systematic review of studies with migrants and refugees from the Middle East and North Africa living in high-income countries found that the prevalence of FI ranged from 40 % to 71 %^(10)^. Other studies found that among Lebanese migrants living in Australia, the prevalence of FI was 72·7 %^(35)^, whereas Haitian migrants residing in Chile had a prevalence of 78 %^(36)^. In the USA, another systematic review on migrants and seasonal agricultural workers showed that the prevalence of FI varied depending on the region where the research was carried out. Thus, it found that the highest rates of FI were in the southwest USA. In Texas and New Mexico, 82 % of those tested had FI, including 49 % who had very poor food security. In contrast, the lowest rates were found in Pennsylvania, where only 8·2 % had FI^(8)^. In England, 95·9 % of migrant households in Birmingham had FI, and 94·6 % of children lived in households with low or very low food security^(37)^. The causes of the variation between the prevalence of FI in this country and that reported in the present study are multifactorial, such as the inclusion of not only migrants but also refugees or seasonal agricultural workers and the use of different instruments and cut-off points to assess FI.

Scarce studies assessing FI in Venezuelan migrants were found. For instance, a study in Trinidad and Tobago tested the validity of the online application of the FIES. Overall, 61·9 % of respondents displayed behaviours characterised as severe FI^(20)^. In addition to using a different scale, this research was conducted in April 2020, during the quarantine of the first wave of the COVID-19 pandemic, which could explain a higher prevalence of severe FI, consistent with the prevalence of FI in countries from Latin America and the Caribbean during that stage of the pandemic^(9)^. During the pandemic, a study conducted in Peru found that the prevalence of FI among Venezuelan migrants was 87·4 %, and 59·5 % of cases were severe. However, since no validated instrument was used, it is not possible to compare our results^(38)^. Another study performed in March 2021 on Venezuelan migrants living in Lima, the capital of Peru, found a prevalence of 63 % of moderate to severe FI. This study measured FI using the FIES, but the sample was non-probabilistic^(39)^. This higher prevalence, compared with our study, maybe because during March 2021, the COVID-19 vaccination campaign was just starting, and pandemic restrictions persisted^(40)^. However, by February and March 2022, these restrictions were being relaxed.

The factors associated with FI vary depending on the setting where it is evaluated. In migrants from the Middle East and North Africa, acculturation and socio-economic factors were associated with FI. Low maternal education, immigration status or logistic difficulties in storing and preparing food were associated with FI among migrants and seasonal agricultural workers in the USA^(8)^. In England, household size, grocery store location and food affordability were related to FI among migrants^(37)^. In Haitian migrants in Chile, having children, limited Spanish proficiency, not having access to basic services and being illegal residents were associated with FI^(36)^. Finally, among Venezuelan migrants in Trinidad and Tobago, FI was less likely among those who were employed and higher among those who paid rent^(20)^. Although we did not measure all the variables included in these studies, similar associations were found in our study. Specifically, unemployment status was found to be associated with all the FI categories (mild, moderate or severe) in Venezuelan migrants residing in Peru, suggesting that some determinants of FI in migrants are similar regardless of the country where they live.

Unemployed Venezuelan migrants are more likely to present mild, moderate or severe FI than those employed. According to ENPOVE-2022, at least three-quarters of Venezuelan migrants were employed. Nevertheless, another survey showed that the income of most of the migrant population is below the minimum vital income, which limits the possibility of accessing housing and other necessities. Unfortunately, 76 % live in a rented room where an average of three people, not necessarily relatives, live^(41)^. In this regard, the association between living with two or more people and having moderate FI reflects the precarious socio-economic conditions that impede migrants from residing in individual dwellings. Similarly, even though those who were never married had a higher prevalence of severe FI, it does not mean that they are exempt from the family burden and the economic responsibilities that this implies. In fact, 66·5 % of Venezuelan migrants in Peru indicated that they were in a cohabiting relationship^(41)^, which implies that they could be included in the group of those who were never married.

Labour informality, which is very frequent in Peru, jeopardises earnings of migrants, making them more susceptible to labour exploitation and other risks. However, while this study did not assess the type of work of the participants, this factor could have several implications. In Peru, 80·8 % of Venezuelan migrants work in informal jobs that do not require specialisation or academic degrees, which suggests that there could be many unemployed migrants with a low socio-economic status despite having higher education^(42)^. In addition, the educational agencies that regulate foreign degrees are highly bureaucratic, which makes it difficult to validate the degrees of migrants in Peru. These facts would explain the potential interaction between education level and employment with FI. On the other hand, although illegal immigration status does not limit finding informal jobs, it does limit obtaining health insurance. Thus, in conditions that require regular therapy for the treatment of chronic diseases, there is little room to provide adequate nutrition. Surprisingly, according to our findings, only 24·15 % of Venezuelan migrants had health insurance. In the distribution of expenses of a Venezuelan migrant, 40 % is allocated to housing rent, 37 % to food and only 2 % to health^(41)^. Consequently, there would be an increase in health expenses if they have a chronic illness, reducing food expenses, which may explain our findings.

There is a culture of discrimination in Peru with a stereotyped image of the Venezuelan population, even in workplaces^(16,41)^. Additionally, most Peruvians believe that Venezuelan refugees and migrants occupy too many job opportunities^(16)^, although Venezuelan are employed primarily in informal jobs^(42)^. In this study, it was found that experiencing perceived discrimination was associated with moderate and severe FI. Venezuelans reported being treated more harshly at work than their Peruvian counterparts, and 32·4 % reported having received unjustified dismissal^(41)^. These discriminatory behaviours jeopardise the continuity of employment of migrants and, thus, their ability to maintain a regular income and enough nutritious food.

The prevalence of FI varied in each city of residence. Tumbes, a border city located in northern Peru, had the highest prevalence of moderate and severe FI and was strongly associated with severe FI. This could be explained by the fact that this city is the main entry point for Venezuelan migrants to Peru. However, it is only a transit city, as most migrants seek to reach Lima to settle. The productive activities or socio-economic characteristics could influence FI in Venezuelan migrants in the cities where they migrate. The probability of mild FI was higher for those living in Ica compared with migrants living in Lima, even when adjusting for multiple potential confounders. For instance, jobs in the agriculture sector predominate in Ica, whereas in Lima, 70 % of migrants are engaged in commerce-related activities^(41)^, being the salary received and the potential job positions possible explanations for this finding. The adaptation of Venezuelan migrants to the employment possibilities of the predominant labour sectors is key. Hence, labour or other dynamics may influence the risk of FI in this population. Further studies are needed to evaluate FI according to the employment characteristics of Venezuelan migrants in these regions.

### Relevance and recommendations for public health

In the general population, FI has a negative impact on health and is correlated with a series of chronic conditions with negative implications not only for individuals but also for society^(4–8)^. In addition, FI-related health complications saturate the healthcare system. Other consequences include loss of productivity and increased inequality^(8)^. These individual and social implications justify establishing governmental strategies to address them.

In Peru, periodic evaluation of FI in migrants is of paramount importance for developing, implementing and supervising nutritional programmes, not only for the migrant population. In migrants, inter-sectoral and multi-sectoral interventions should be focused on the characteristics associated with FI. For example, in the USA, in addition to improving eating habits, it is possible to access federal assistance programmes (free or reduced-price school lunches) through the National School Lunch Program or the Nutrition Assistance Program^(8)^. In Trinidad and Tobago, the United Nations High Commissioner for Refugees operates an intervention system that helps migrants with money for food^(20)^. Other organisations, such as the International Organisation for Migration and the Venezuelan Solidarity Network of Trinidad and Tobago, also organise distribution campaigns to provide food baskets to migrants^(20)^. Although there are initiatives that provide emergency assistance to vulnerable and at-risk Venezuelan migrant children and families to cover their basic needs in Peru^(43)^, they are not government programmes. In this sense, considering that the Venezuelan community in Peru is now a permanent community, they should be included in nutritional support programmes.

### Limitations and strengths

This study has some limitations. First, due to the cross-sectional design of this study, causality cannot be determined. However, given the nature of the outcome and the independent variables, reverse causality is unlikely. Second, since this study was based on an analysis of secondary data, it was subjected to the variables collected in the survey without considering others that would be of interest to assess, such as those related to acculturation and logistical difficulties in acquiring, storing and preparing food. Third, social desirability and memory biases could be present. Nevertheless, most of the questions were related to recent events, and well-trained pollsters collected the information. Despite these limitations, this study has several strengths. The probabilistic sample design provides national estimates with the representation of most of the Venezuelan migrants living in Peru. Moreover, a validated and equated scale – with adequate psychometric properties – was used to measure FI, allowing comparison with other studies. Furthermore, all the levels of the FIES were considered in the analysis. According to Pérez-Escamilla *et al.*, this approach is pivotal for addressing and assessing public policies and programmes, as it identifies the dose–response or curvilinear relationships under assessment^(44)^. Taking all of this into account, this study provides an overview of the determinants of FI in Venezuelan migrants residing in Peru, which enables the identification of high-risk groups in which governmental strategies should be targeted.

## Conclusion

In Peru, four out of ten Venezuelan migrants presented moderate to severe FI in 2022. The FIES showed adequate psychometric properties. Several socio-demographic, health status and migratory characteristics were found associated with the different degrees of FI. Since the Venezuelan community in Peru is now a permanent community, inter-sectoral and multi-sectoral interventions are needed and should be focused on addressing the determinants of FI.

## Supporting information

Al-kassab-Córdova et al. supplementary material 1 Al-kassab-Córdova et al. supplementary material

Al-kassab-Córdova et al. supplementary material 2 Al-kassab-Córdova et al. supplementary material

## Supplementary material

For supplementary material accompanying this paper visit https://doi.org/10.1017/S1368980023002513
