# Supplementary figures and images for: Socio-demographic, migratory and health-related determinants of food insecurity among Venezuelan migrants in Peru
Source: Public Health Nutr. 2023 Nov 10;26(12):2982–94. doi: 10.1017/S1368980023002513 (PMC10755391; doi:10.1017/S1368980023002513)

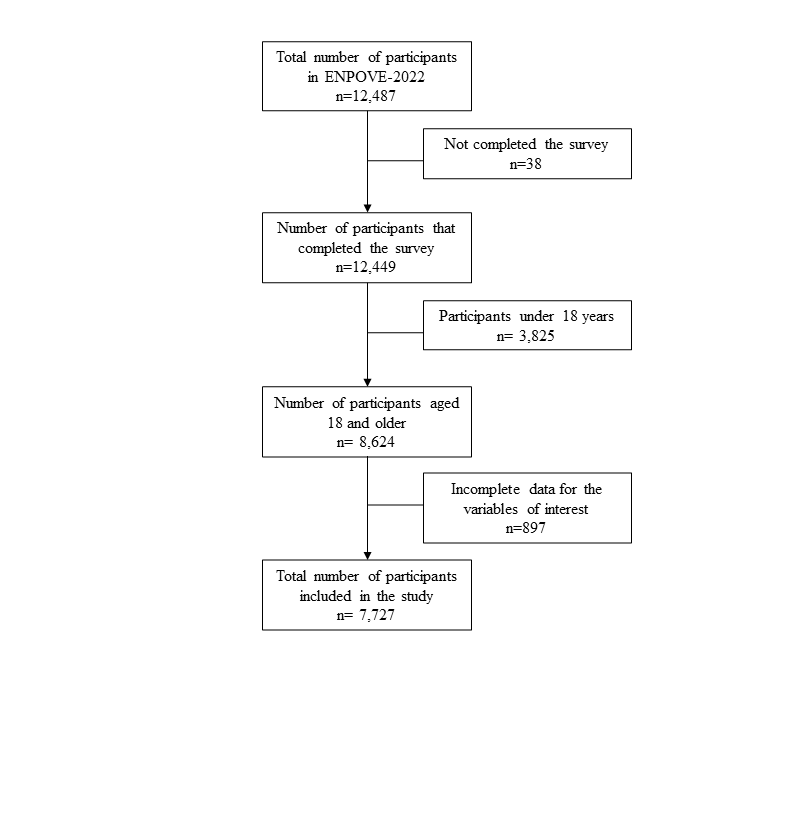

Supplement: Al-kassab-Córdova et al. supplementary material 1 — Al-kassab-Córdova et al. supplementary material [file S1368980023002513sup001.tif]
