# Supplementary material for: Socio-demographic, migratory and health-related determinants of food insecurity among Venezuelan migrants in Peru
Source: Public Health Nutr. 2023 Nov 10;26(12):2982–94. doi: 10.1017/S1368980023002513 (PMC10755391; doi:10.1017/S1368980023002513)
Supplement: Al-kassab-Córdova et al. supplementary material 2 — Al-kassab-Córdova et al. supplementary material [file S1368980023002513sup002.docx]

**Supplementary 3.** Items used for equating

| **Raw score** | **Respondent severity parameter** | **Measurement error** | **Weighted percent of cases with each raw score** | **Probability of food insecurity (moderate to severe level)*** | **Probability of food insecurity (severe level) *** |
| --- | --- | --- | --- | --- | --- |
| 0 | -3.48196742 | 1.572383 | 0.03731881 | 0.0% | 0.0% |
| 1 | -2.55806627 | 1.2074762 | 0.09049411 | 0.6% | 0.0% |
| 2 | -1.41499728 | 0.9451045 | 0.10675102 | 2.3% | 0.0% |
| 3 | -0.61721222 | 0.8453182 | 0.11275727 | 10.0% | 0.0% |
| 4 | 0.04571223 | 0.816416 | 0.10883319 | 30.3% | 0.1% |
| 5 | 0.71006759 | 0.8352196 | 0.10210619 | 61.4% | 1.6% |
| 6 | 1.4579407 | 0.9129993 | 0.10194602 | 86.1% | 12.7% |
| 7 | 2.48152808 | 1.1441547 | 0.15872507 | 96.1% | 49.4% |
| 8 | 3.34547873 | 1.572383 | 0.18106831 | 96.6% | 70.5% |
| Notes: * Probabilities are based on the thresholds used by FAO to classify respondents into categories of food insecurity. | | | | | |
